# Synergistic antitumor effect of HMGB2 targeting combined with MDM2 inhibitor in diffuse intrinsic pontine glioma therapy

**DOI:** 10.3389/fonc.2026.1867524

**Published:** 2026-09-15

**Authors:** Bochao Zhang, Yuxuan Deng, Luyang Xie, Yiying Mai, Hang Zhou, Liwei Zhang

**Affiliations:** Department of Neurosurgery, Beijing Tiantan Hospital, Capital Medical University, Beijing, China

**Keywords:** combined therapy, DIPG, HMGB2, inflachromene, MDM2 inhibitor, tumor suppressor p53

## Abstract

Diffuse intrinsic pontine glioma (DIPG) is a brain malignancy in children with a dismal prognosis. The high mobility group box 2 (HMGB2) is a member of the HMGB family of chromatin-associated proteins, and is identified as a potential oncogene in various cancers. However, the effects of HMGB2 in DIPG remains unclear. In this study, we observed aberrant expression of HMGB2 in multiple cancer types, with its expression level being negatively correlated with clinical outcomes across pan-cancers. High expression of HMGB2 was determined in DIPG cells, but not in primary brainstem neural progenitor cells (PPCs). Knockdown of HMGB2 significantly inhibited the DIPG cell proliferation and induced cell apoptosis. Intriguingly, bulk RNA sequencing revealed that inhibition of HMGB2 upregulated p53 protein expression and enhanced the activity of p53-p21 axis. Combining HMGB2 inhibitor with a p53-MDM2 inhibitor RG7388 resulted in a synergistic suppression of DIPG cell growth and promoted cell apoptosis by activating the tumor suppressor p53 and p21 expressions *in vitro*. The animal studies demonstrated that the combination treatment significantly inhibited tumor growth and prolonged the survival of mice bearing orthotopic DIPG tumors. These findings demonstrate that HMGB2 acts as an oncogene in DIPG tumorigenesis, and targeting HMGB2 combined with MDM2 inhibitor provides a novel preclinical basis for the development of DIPG therapeutic strategies.

## 1 Introduction

Diffuse intrinsic pontine glioma (DIPG), a subtype of diffuse midline glioma (DMG), is a pediatric glioma that accounts for 80% of all brainstem gliomas and is the primary cause of death related to brain tumors in children (1, 2). DIPG occurs at a rate of 1.78 per 100, 000 individuals, with the highest incidence observed between ages 6 and 9. The median survival time for DIPG is under one year, and the five-year survival rate is below 5% (3, 4). Radiation therapy, the standard of care as outlined by the NCCN Guidelines (5), can extend survival by only 3 to 4 months. Other management strategies, aside from local irradiation, have proven ineffective (6, 7).

Recent advances have greatly enhanced our understanding of DIPG biology. These tumors are mainly defined by mutations in the H3.3 and H3.1 genes (1, 8). In the 2021 WHO classification, DMGs were reclassified as those with H3 K27 alterations (9). Other common genetic alterations in DIPG, such as mutations in tumor protein p53 (TP53), epidermal growth factor receptor (EGFR), protein phosphatase 1D (PPM1D), and activin A receptor type 1 (ACVR1), have been identified as potential therapeutic targets (10). These discoveries have revealed the molecular basis of the disease and opened up possibilities for targeted therapies. However, despite ongoing efforts, the prognosis for DIPG has remained largely unchanged for decades, highlighting the urgent need for improvements in treatment outcomes.

The high mobility group box (HMGB) family is the second most abundant chromatin protein family in eukaryotic cells, and is involved in repairing damaged DNA and regulating DNA replication, transcription, and recombination (11). Among these, HMGB2 has been implicated in tumorigenesis in various cancers (12, 13). Elevated expression of HMGB2 has been associated with decreased survival of patients with breast and gastric cancer and acts as an anti-apoptotic factor in cell proliferation and invasion (14, 15). However, the role of HMGB2 in DIPG remains poorly understood. In the current study, we aimed to investigate the functions of HMGB2 in DIPG and explored its potential as a therapeutic target, particularly in combination with other drugs, to provide a new preclinical perspective for tackling this devastating disease.

## 2 Material and methods

### 2.1 Cell culture

The patient-derived DIPG cell lines were obtained and cultured as previously reported (16). Briefly, DIPG cells were preserved in DMEM medium supplemented with the B-27 serum-free supplement, basic fibroblast growth factor (bFGF), epidermal growth factor (EGF), platelet-derived growth factor-AB (PDGF-AB) and 1% penicillin/streptomycin. Primary brainstem neural progenitor cells (PPCs) were maintained in the above medium, excluding PDGF-AB, which were immature neural precursor cells derived from normal brainstem tissue. Sharing the same tissue origin and developmental background as diffuse intrinsic pontine glioma (DIPG), a tumor arising from brainstem neural precursor cells, PPCs can minimize interference from tissue-specific differences on experimental results, making them an ideal control for verifying tumor cell-specific molecular abnormalities. PPCs were isolated from the brainstem tissue of donors without neurological diseases, and the isolation region was consistent with the predilection site of DIPG (pons). Single-cell suspensions were obtained by mechanical trituration combined with enzymatic digestion (trypsin + DNase I), and neural progenitor cells were purified via density gradient centrifugation (Percoll gradient). Immunofluorescence staining was performed to verify the expression of neural progenitor cell markers (Nestin-positive, SOX2-positive), while confirming low expression of mature neuronal marker (β-III Tubulin) and astrocyte marker (GFAP), ensuring a cell purity of > 90%. Cell cycle analysis was conducted by flow cytometry to validate that their proliferative activity was consistent with the characteristics of primary neural progenitor cells. All cells were cultured in an incubator at 37 °C, and were tested negative for mycoplasma prior to conducting further experiments. All experiments with human tissues were performed with ethical approval of the Institutional Review Board of Beijing Tiantan Hospital, Capital Medical University (Approval NO. KY 2018-042-02), all methods were in accordance with the Declaration of Helsinki. All human tissues were obtained with written informed consent. The three DIPG cell lines (TT150714, TT150630, TT170720) used in this study were confirmed to carry wild-type TP53 genotype via previous genotyping identification in our laboratory.

### 2.2 The expression of HMGB2 in human cancers

The expression of HMGB2 in normal tissues and counterpart tumor from different cancer types was assessed in the Gene Expression Profiling Interactive Analysis (GEPIA) database (http://gepia.cancer-pku.cn/), and the Kaplan-Meier Plotter software (http://kmplot.com/analysis/) was used to analyze the association between HMGB2 expression and clinical outcomes in pan-cancer patients.

### 2.3 Lentivirus-mediated transfection

To generate HMGB2 stable knockdown cells, shHMGB2 sequences were cloned into a pLV-hU6-shRNA-puro vector to create shHMGB2-expressing construction. Then the resulting vector was co-transfected with psPAX2 and pMD2G into HEK293T cells. After 48 hours, the lentiviral supernatants were collected. DIPG cells were then treated with lentiviral particles in 2-10 μg/mL polybrene for 48 hours. Following infection, puromycin (0.5-2 µg/mL) was applied to select for stable HMGB2 knockdown cells. The knockdown efficiency of HMGB2 in DIPG cells was verified by Western blotting prior to conducting further experiments. The primer of shRNA was shown as below: shHMGB2#1, Forward 5’-GCTCAATACTAGCTTCAGTAT, Reverse 5’-ATACTGAAGCTAGTATTGAGC; shHMGB2#2, Forward 5’-GCAAAGGAGAAGTCGAAGTTT, Reverse 5’-AAACTTCGACTTCTCCTTTGC; shHMGB2#3, Forward 5’-GGTGGACGCGGATCTGTCAAC, Reverse 5’-GTTGACAGATCCGCGTCCACC.

### 2.4 Bulk RNA sequencing

The bulk RNA sequencing analysis was conducted as previously reported (16). Briefly, DIPG cells were treated with a HMGB2 inhibitor Inflachromene (ICM) for 24 hours. Then the total RNA was isolated from the treated cells using the Total RNA Purification Kit (catalog no. 8034111, DAKEWE, China) following the manufacturer’s instructions. Then the resulting RNA was resuspended in nuclease-free water, and RNA sequencing was conducted on the Illumina HiSeq X platform (Novogen). The raw sequence data reported in this paper have been deposited in the Genome Sequence Archive (Genomics, Proteomics & Bioinformatics 2021) in National Genomics Data Center (Nucleic Acids Res 2024), China National Center for Bioinfomation/Beiling Institute of Genomics, Chinese Academy of Sciences (GSA: subHRA015865) that are publicly accessible at https://ngdc.cncb.ac.cn/gsa (17, 18).

### 2.5 Cell viability assay

The cell viability assays were performed after HGMB2 knockdown. The shNC and shHMGB2 DIPG cells were harvested and seeded in a 96 well plate with 5000 cells per well, and cultured for 24 hours for adhesion. For the combined treatment, the shNC and shHMGB2 DIPG cells were treated with 20 nM of RG7388, a selective p53-MDM2 inhibitor, for 24 hours. Alternatively, DIPG cells were treated with increasing concentrations of ICM or HMGB2 alone or the combination of these two drugs for up to 96 hours. For routine functional experiments, ICM was used at 500 nM and RG7388 was used at 20 nM. Gradient concentrations ranging from 25 nM to 6400 nM were applied for both ICM and RG7388 in drug combination and synergism analyses. The concentration settings were determined based on the IC_50_ values of ICM in tested DIPG cell lines and previously published literature (19). Cell proliferation was assessed through quantification of adenosine triphosphate (ATP) (CellTiter-Glo Luminescent Reagent, Promega) according to the manufacturer’s protocol. Briefly, Celltiter-Glo reagent was added to each well, and the plates were then incubated at 37 °C for 15 min. Then luminescence was recorded on a luminescence microplate reader.

The expected effect value was calculated using the Bliss independence model with the formula: E expected= EA + EB – EA × EB (where E expected= expected inhibition rate, EA and EB = actual inhibition rates of Drug A and Drug B alone, respectively). The synergistic effect was quantitatively verified by comparing the difference between the expected effect and the actual observed effect. If the actual effect was significantly higher than the expected effect (P<0.05), the synergistic relationship was further confirmed. With the aid of CompuSyn software, the Combination Index (CI) under different concentration combinations was calculated based on the drug dose-effect curves. A CI value < 1 indicates a synergistic effect, CI = 1 indicates an additive effect, and CI > 1 indicates an antagonistic effect. The CI values of representative dose combinations were 0.62 ± 0.05 (TT150714 cells, P<0.01) and 0.58 ± 0.04 (TT150630 cells, P<0.001) for ICM 500nM + RG7388 20nM, with statistical significance analyzed by one-way ANOVA.

### 2.6 Flow cytometry analysis

DIPG cells were harvested and placed in a 24 well plate with 2x10^4^ per well and cultured overnight for adhesion. Then the cells were treated with RG7388 or HGMB2 inhibitor alone or the combination of these two drugs for 24 hours. Cell apoptosis assay was performed by using Annexin V/FITC-propidium iodide (PI) double staining Kit (BD Pharmingen, catalog no. 556547). Following cell transfection, the DIPG cells were successively stained with Annexin V/FITC and PI following the manufacturer’s protocol. After that, the resulting cells were analyzed for apoptosis using FACS Calibur flow cytometry (BD Biosciences), with data processed in FlowJo v.10 software.

### 2.7 Cell cycle analysis

DIPG cells were harvested and placed in a 12-well plate with 5x10^5^ per well and cultured overnight for adhesion. Then the cells were treated with RG7388 or HGMB2 inhibitor alone or the combination of these two drugs for 24 hours. Cell cycle analysis was performed using the Elabscience^®^ Cell Cycle Assay Kit (Red Fluorescence) according to the manufacturer’s protocol. Briefly, the cells were collected and 100 μL of RNase A reagent was added for 30 mins incubation at 37°. Then 50 μg/mL of PI was added and incubated in the dark at 4 °C for 30 mins. After that, the resulting cells were analyzed for cell cycle analysis using FACS Calibur flow cytometry, with data processed in FlowJo v.10 software.

### 2.8 Western blotting

The cells or tissues were lysed with RIPA buffer together with phosphatase and proteinase inhibitor cocktails, and protein extracts were quantitated by BCA assay following the manufacturer’s protocol. Approximately 10-20 μg of protein was loaded onto an 8%-12% SDS/PAGE, electrophoresed, and transferred to a PVDF membrane. No pre-cutting was performed on the membrane before antibody hybridization, and all blots were detected on the complete membrane before cropping as needed. After blocking with 5% milk in Tris-buffered saline (TBS) with 0.1% Tween 20, the membrane was incubated with primary and secondary antibodies. Primary antibodies: Anti-HMGB2 (Proteintech, Cat# 14597-1-AP), Anti-p53 (Proteintech, Cat# 10442-1-AP), Anti-p21 (Abcam, Ab188224), Anti-GAPDH(Proteintech, Cat# 10494-1-AP), Anti-β-actin (Proteintech, Cat# 20536-1-AP); Secondary antibodies: HRP-conjugated goat anti-rabbit IgG (Proteintech, Cat# SA00001-2) and HRP-conjugated goat anti-mouse IgG (Proteintech, Cat# SA00001-1). All antibodies were used at the dilution ratio of 1:1000 for primary antibodies and 1:5000 for secondary antibodies. Immunocomplexes were detected using the Syngene Gbox XX9, and the images were analyzed using FIJI-ImageJ software. Original unprocessed blots with membrane edges and molecular weight ladders are provided in **Supplementary File**.

### 2.9 Immunohistochemical staining

The tissues were fixed in 4% paraformaldehyde (PFA) for 48 hours. After dehydration through a series of ethanol, the tissues were embedded in paraffin blocks, and the specimens were sectioned into 5 μm slices using a Leica RM 2235 rotary microtome (Leica Biosystems). Then the slides were transferred to 100%, 95%, 70%, and 50% ethanol, and soaked for 5 minutes before proceeding with immunohistochemical (IHC) staining of p21 (anti-p21 antibody, Abcam, catalog no. Ab188224).

### 2.10 Animal study

The animal experiments were carried out in accordance with the National Research Council’s Guide for the Care and Use of Laboratory Animals and the protocol (#202202008) was approved by the Institutional Animal Care and Use Committee of Capital Medical University. Based on the pre-experiment results, the sample size was calculated by GraphPad Prism 9.0 with α=0.05, power=80% and intergroup difference=30%, and 5 mice were assigned to each group. The 4-week-old female NCG (NOD/ShiLtJGpt-*Prkdc^em26Cd52^Il2rg^em26Cd22^*/Gpt) mice was obtained from GemPharmatech Co., Ltd (Jiangsu, China), and kept in a specific pathogen-free (SPF) environment. Mice were randomly assigned to four experimental groups (Control group, ICM group, RG7388 group, ICM + RG7388 combined group) using a random number table right after successful orthotopic DIPG tumor models confirmation. Researchers responsible for tumor imaging, measurement and survival recording remained blinded to group assignments throughout the entire experiment to avoid subjective bias.

The orthotopic DIPG models were generated by implanting luciferase-transduced DIPG cells into the pons of NCG mice. Briefly, mice were anesthetized with 2% isoflurane in oxygen by inhalation, and then approximately 1 × 10^5^ DIPG cells suspended in 5 μL of saline were injected into the pons of anesthetic mice at an injected rate of 2.5 uL/min controlled by Nanoject III Programmable Nanoliter Injector. After orthotopic injection of luciferase-expressing DIPG cells into the mouse pons, we performed routine *in vivo* bioluminescence imaging on day 7 post-implantation via the IVIS system to confirm successful tumor engraftment in all mice. Only mice with detectable bioluminescent signals in the pontine region confirmed by IVIS imaging were enrolled into formal treatment groups; mice without obvious tumour signals were excluded from subsequent experiments to eliminate bias. Then treatment with HMGB2 inhibitor ICM (10 mg/kg), MDM2 inhibitor RG7388 (50 mg/kg), ICM combined with RG7388, or vehicle was started daily after one week after implantation. The dose of ICM (10 mg/kg/day) was selected according to previous study on mouse central nervous system disease models (20). The dose of RG7388 (50 mg/kg/day) was chosen based on published brain tumor xenograft studies and our in-house pre-experimental results (21). During the whole treatment period, the body weight, food intake and daily activity of mice were recorded every day. No obvious weight loss, behavioral abnormality or drug-related death was observed in each group. The tumor growth rate was monitored by bioluminescent imaging. The tumor growth rate was monitored by bioluminescent imaging, and IVIS imaging data were shown in **Supplementary File**. All mice were sacrificed via cervical dislocation immediately once they reached the pre-defined humane euthanasia endpoints (including severe weight loss >20%, limb paralysis, inability to feed/drink, or pre-set maximum experimental time limit of 50 days). Tumor tissues were dissected and processed within 10 minutes after euthanasia. No scheduled mid-treatment tissue collection was performed in this study. All tissue samples were collected uniformly at the experimental endpoint across four groups. Harvested tumor tissues were split into two parts: one portion was immediately placed in 4% paraformaldehyde for 48 h fixation for subsequent IHC staining; the other portion was snap-frozen in liquid nitrogen and stored at −80 °C for Western blot protein extraction. Since all tissues were collected under identical timing and processing conditions at the unified endpoint, the inter-group comparability of IHC and Western blot results is guaranteed. No time-dependent degradation or processing bias existed among groups. The integrated optical density (IOD) of p21 positive staining was calculated using ImageJ software. All WB bands (HMGB2, p53, p21) from tumor tissues were quantified via ImageJ grey value analysis.

### 2.11 Statistical analysis

All data were expressed as the mean ± standard deviation (SD). Statistical analysis was conducted using GraphPad Prism 9.0 software. For comparison between two groups, unpaired two-tailed Student’s t-test was applied. For comparison among three or more groups, one-way analysis of variance (one-way ANOVA) was performed firstly, followed by *post-hoc* multiple comparison tests. Sample size calculation was performed based on the pre-experiment results: at least 3 technical replicates and 3 biological replicates for *in vitro* experiments; n=5 per group for *in vivo* animal experiments. A P value < 0.05 was considered statistically significant.

## 3 Results

### 3.1 DIPG cells had a high expression level of HMGB2

We first analyzed the mRNA expression levels of HMGB2 across various human cancers and paired normal samples using the GEPIA database, which includes data from both TCGA and GTEx. In the RNA-seq data from TCGA datasets, HMGB2 was found to be overexpressed in several cancer types, including adrenocortical carcinoma (ACC), bladder urothelial carcinoma (BLCA), colon adenocarcinoma (COAD), lymphoid neoplasm diffuse large B-cell lymphoma (DLBC), glioblastoma multiforme (GBM), and thymoma (THYM) (**Figure 1A**). To assess potential association between HMGB2 expression level and clinical outcomes of patient across different cancer types, we conducted a survival analysis using the KM plotter database. As shown in **Figure 1B**, enhanced expression of HMGB2 was significantly related with poor prognosis in pan-cancers. To investigate the HMGB2 expression in DIPG, we analyzed different DIPG cell lines using Western blotting. The results indicated that the DIPG cell lines TT150714, TT150630, TT170720, TT190313, and TT190326 exhibited significantly higher HMGB2 expression compared to primary brainstem neural progenitor cell lines, PPC-1 and PPC-2 (**Figure 1C**). These results suggested HMGB2 may act as an oncogene in DIPG tumorigenesis and is highly expressed in DIPG.

**Figure 1:**
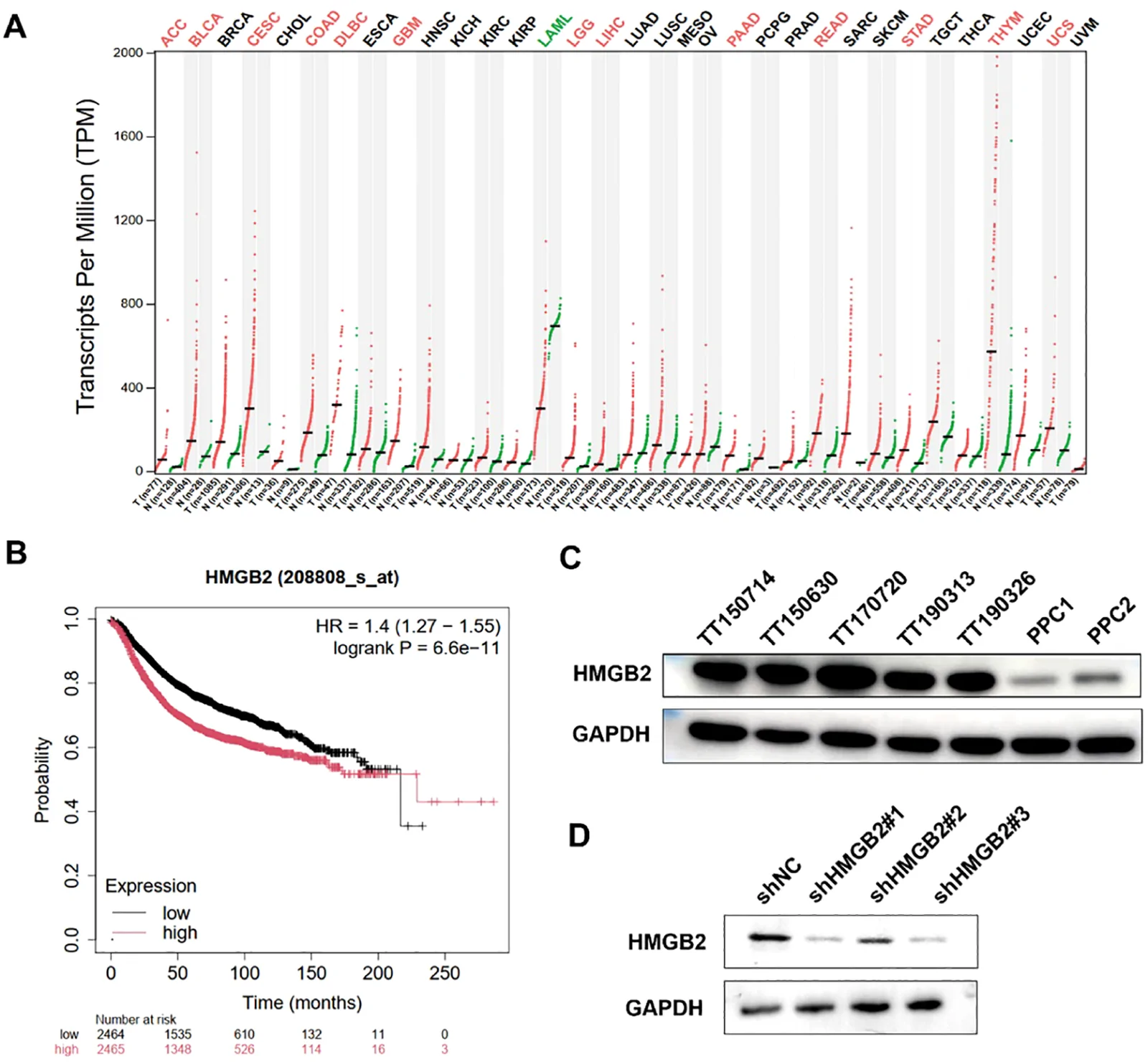
HMGB2 expression in different cancer types and its association with the prognosis of patients. **(A)** The mRNA expression levels of HMGB2 in the patients with pan-cancers using GEPIA database. **(B)** Association between HMGB2 expression and prognosis of the patients with pan-cancers. **(C)** HMGB2 expression level in different cell lines by western blot analysis. **(D)** Down-regulation of HMGB2 by lentivirus-mediated RNAi transduction and the HMGB2 expression was verified by western blot analysis.

### 3.2 Knockdown of HMGB2 enhances the p53 expression level in DIPG cells

To investigate the function of HMGB2 *in vitro*, we down-regulated its expression in DIPG cell lines using lentivirus-mediated shRNA interference. Western blot analysis confirmed efficient knockdown of HMGB2 expression with shHMGB2#1 and shHMGB2#3 (**Figure 1D**). These two shRNAs were used for further experiments. As shown in **Figures 2A–C**, knockdown of HMGB2 expression was observed in TT150714, TT150630, and TT170720 cell lines using shHMGB2#1 and shHMGB2#3. Next, bulk RNA sequencing was performed on DIPG cells after treatment with a HMGB2 inhibitor Inflachromene (ICM) (22). The differential expression of upregulated and downregulated genes in DIPG cells after HMGB2 inhibition by ICM treatment was shown in **Figure 2D**, with HMGB2 notably downregulated. KEGG pathway analysis of RNA sequencing data revealed that the p53 signaling pathway was significantly impacted following HMGB2 inhibition (**Figures 2D–F**). The tumor-suppressor protein p53, or guardian of the genome, regulates key biological responses, including DNA repair, cell cycle arrest, and apoptosis (23, 24). We further examined p53 expression in HMGB2 knockdown cells and found a significant increase in p53 levels (**Figures 2A–C**), consistent with data obtained from RNA sequencing (**Figure 2D**). Collectively, these data suggested inhibition of HMGB2 is associated with activation of tumor-suppressor protein p53 in DIPG cells.

**Figure 2:**
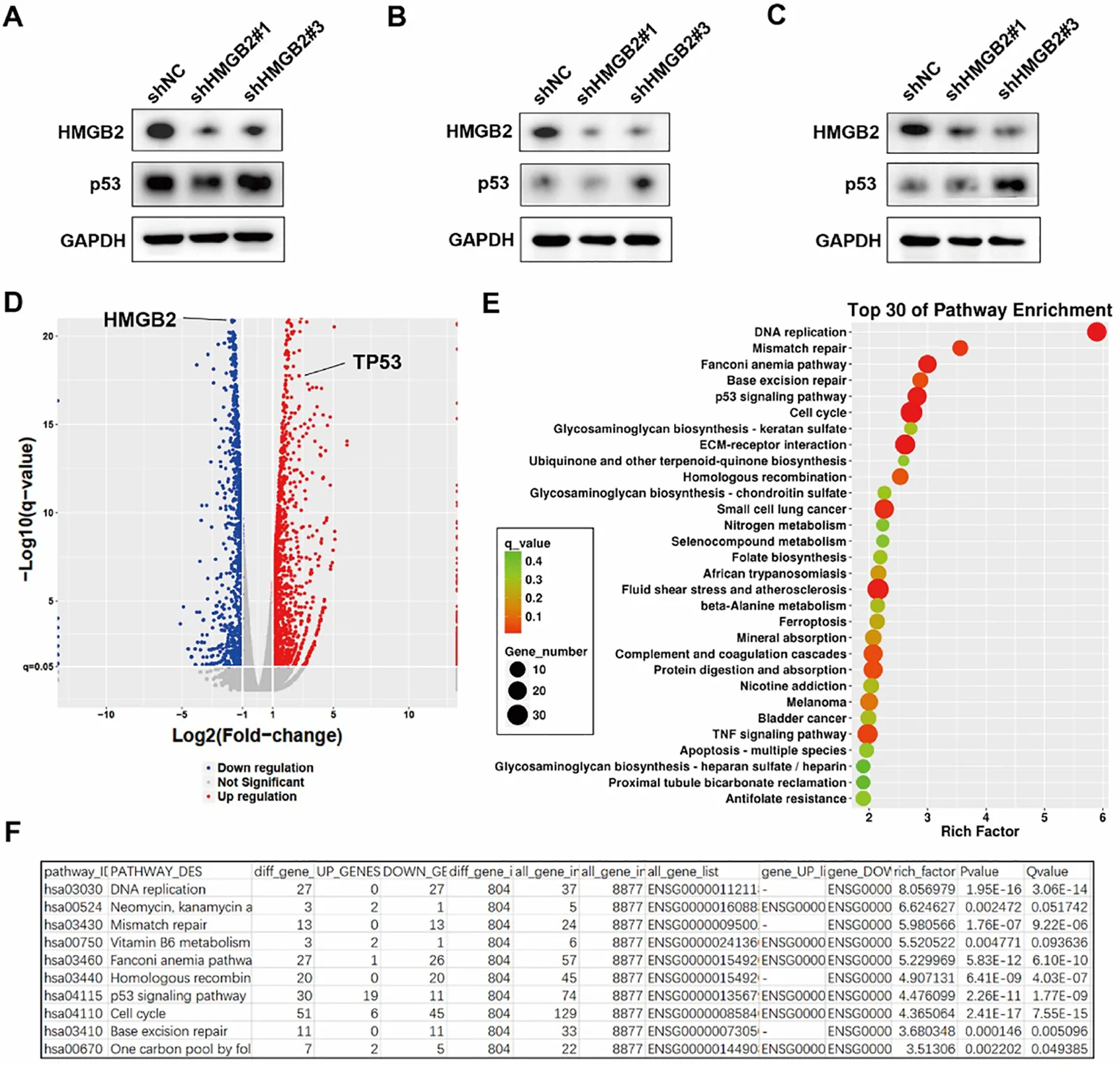
Inhibition of HMGB2 was associated with activation of p53 in DIPG cells. **(A-C)** Western blot analysis of HMGB2 and p53 expression level after HMGB2 knockdown by lentivirus-mediated shRNA interference in TT150714 **(A)**, TT150630 **(B)**, TT170720 **(C)** cell lines. **(D)** Volcano plots of up-regulated and down-regulated differential expressed genes in DIPG cells after HMGB2 inhibition by ICM treatment. **(E)** Top 30 of pathway enrichment by a KEGG pathway-based RNA sequencing. **(F)** The involved genes from the top 10 of signaling pathway showed in **(E)**.

### 3.3 HMGB2 inhibition combined with MDM2 inhibitor synergistically inhibits DIPG cell growth

The effects of HMGB2 on DIPG cell growth were validated through cell viability assays. Knockdown of HMGB2 significantly inhibited cell growth in the TT150714, TT170720 and TT150630 cell lines (**Figures 3A–C**). To explore the potential benefits of combining HMGB2 inhibition with other drugs, we treated HMGB2 knockdown cells (shHMGB2#1 and shHMGB2#3) with a p53-MDM2 inhibitor RG7388 (25), and found that RG7388 alone significantly inhibited DIPG cell growth. Moreover, it enhanced the inhibitory effects of HMGB2 knockdown induced by shHMGB2#1 and shHMGB2#3 (**Figures 3A–C**). Furthermore, we combined the HMGB2 inhibitor (ICM) and RG7388 in treatment of TT150714 and TT150630 cell lines. The IC50 of ICM was 531 nM and 468 nM for TT150714 and TT150630 cells, respectively. Accordingly, cells were treated with increasing concentrations of ICM (ranging from 25 nM to 6400 nM) in combination with RG7388 at different concentrations (25 nM to 6400 nM). A synergistic analysis showed that the combined treatment exhibited significant synergistic effects, with the CI values of representative dose combinations as follows: ICM 250nM + RG7388 20nM, TT150714 (0.75, 95%CI: 0.68-0.82), TT150630 (0.71, 95%CI: 0.65-0.77); ICM 500nM + RG7388 20nM, TT150714 (0.62, 95%CI: 0.56-0.68), TT150630 (0.58, 95%CI: 0.52-0.64); all CI values were < 1 (P<0.05), confirming a strong synergistic interaction between ICM and RG7388 (**Figures 3D–G**). These results indicate the critical role of HMGB2 in cell proliferation, and its inhibition in combination with RG7388 significantly enhances the suppression of DIPG cell growth.

**Figure 3:**
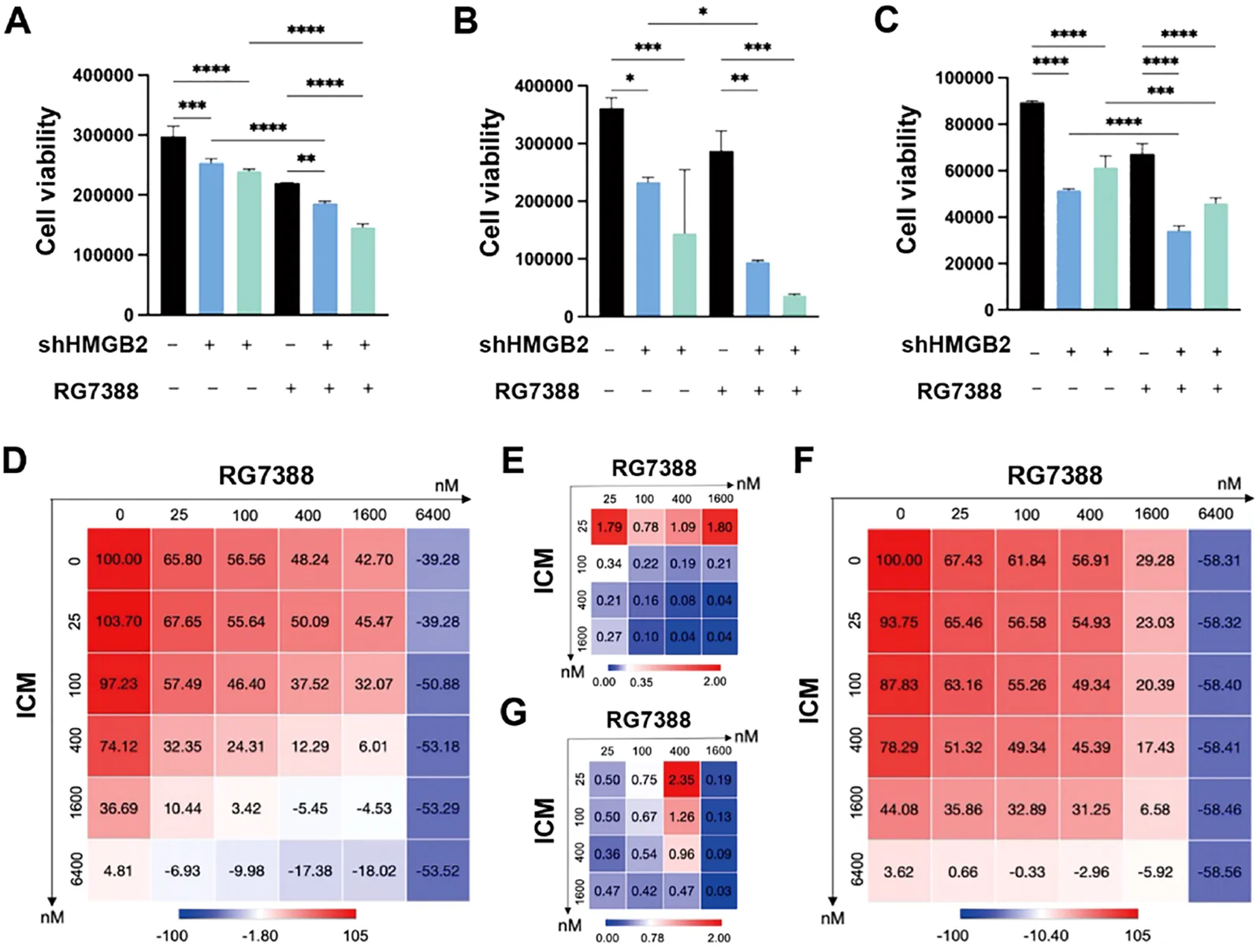
Inhibition of HMGB2 combined with MDM2 inhibitor synergistically suppresses DIPG cell growth. **(A-C)** Cell viability of TT150714 **(A)**, TT170720 **(B)**, and TT150630 **(C)** cells after HMGB2 knockdown by lentivirus-mediated shRNA interference (shHMGB2#1 and shHMGB2#3) in presence or absence of RG7388 treatment. **(D-G)** The cell viability assay was conducted in TT150714 **(D)** and TT150630 **(F)** cells treated with ICM and RG7388, and the combinatory effects were analyzed by synergism analysis in TT150714 **(E)** and TT150630 **(G)** cell lines. One-way ANOVA followed by *post-hoc* multiple comparison tests; *P < 0.05, **P < 0.01, ***P < 0.001, ****P < 0.001.

### 3.4 Inhibition of HMGB2 enhances the cell apoptosis and induces G0/G1 arrest in DIPG cell

We assessed the longitudinal effects of combined treatment in DPIG cells using HMGB2 inhibitor ICM and MDM2 inhibitor RG7388. As shown in **Figure 4A**, treatment with either the ICM or RG7388 alone significantly inhibited DIPG cell growth after 48 hours. However, the combination of both agents showed the strongest inhibitory effect on cell growth (**Figure 4A**). Additionally, an Annexin V/FITC-PI double staining assay revealed an increased apoptotic cell ratio following treatment with the ICM and RG7388. The combination treatment resulted in the highest rate of apoptosis among all groups (**Figures 4B, C**), which was consistent with the growth inhibition data shown in **Figure 4A**. Flow cytometry analysis further revealed that both ICM or RG7388 alone significantly induced G0/G1 cell cycle arrest in DIPG cells, while their combination exhibited a synergistical effect on G0/G1 arrest (**Figure 4D**). Accumulating evidence has demonstrated that p21, a downstream target of p53, plays a key role in inducing G1 cell cycle arrest, which subsequently promotes cell apoptosis (24, 26). Co-treatment of DIPG cells with ICM and RG7388 resulted in the highest expression levels of p53 and p21 compared to the individual treatments or control groups (**Figure 4E**), corroborating the data presented in **Figure 4B, C**. Collectively, these results suggest that the HMGB2 inhibitor, in combination with MDM2 inhibitor, significantly suppresses cell proliferation by activating p53/p21 axis, thereby promoting cell apoptosis *in vitro*.

**Figure 4:**
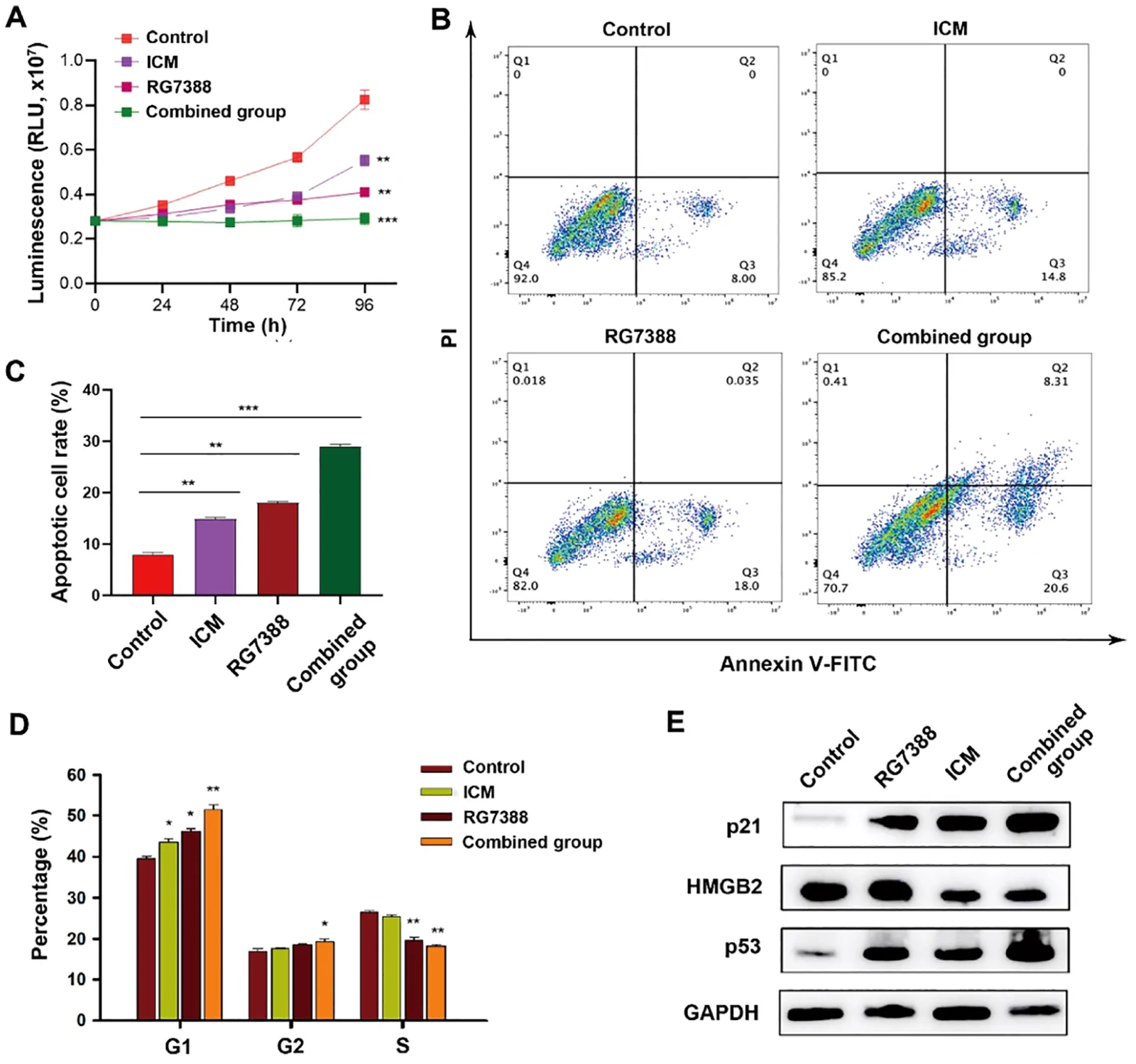
HMGB2 influences the cell proliferation and apoptosis of DIPG cells. **(A)** The cell proliferation assay was conducted in DIPG cell line with different treatment conditions. Student’s t-test; **P < 0.01, ***P < 0.001. **(B)** Representative flow cytometry images for the detection of apoptosis of DIPG cells with different treatment conditions using Annexin V/FITC-PI double staining. **(C)** Quantification of apoptotic cells. Student’s t-test; **P < 0.01, ***P < 0.001. **(D)** Cell cycle analysis of DIPG cells with different treatment conditions. One-way ANOVA followed by *post-hoc* multiple comparison tests; *P < 0.05, **P < 0.01. **(E)** Western blot analysis of DIPG cells with different treatment conditions.

### 3.5 Inhibition of HMGB2 suppresses DIPG tumor growth *in vivo*

Inspired by the *in vitro* data, we evaluated the *in vivo* effects of combining the HMGB2 inhibitor with RG7388 on DIPG tumor pathogenesis. An orthotopic DIPG model was established in NCG mice by implanting luciferase-transduced DIPG cells into the pons of mice. Tumor presence was confirmed through luciferase imaging, and mice with orthotoptic DIPG tumors were treated with either the ICM (10 mg/Kg/day) and/or RG7388 (50 mg/Kg/day). On day 21 post-implantation, bioluminescent imaging showed that tumor growth was faster in the vehicle-treated group compared to either drug treatment group, while no significant difference was observed between the ICM and RG7388 treatment groups (**Figures 5A, B**). the combination treatment group exhibited the slowest tumor growth, highlighting the synergistic inhibitory effects of the two agents on DIPG tumor growth (**Figures 5A, B**). Furthermore, the combination of ICM and RG7388 resulted in prolonged survival in the DIPG mouse model, although no statistically significant survival benefit was observed with RG7388 alone (**Figure 5C**). Tumor tissues were harvested at the experimental endpoint for Western blot analysis and immunohistochemical staining. The results revealed findings consistent with data showed above, including HMGB2 downregulation and p53 and p21 upregulation by small molecular inhibitors (**Figures 5D, E**) (**Supplementary Figure 1**). Overall, the combined strategy resulted in survival prolongation that was not achieved by either drug alone, suggesting that HMGB2 is crucial for DIPG proliferation and tumorigenesis, and thus targeting HMGB2 may serve as a potential therapeutic strategy, particularly in combination with MDM2 inhibitor, in treatment of DIPG.

**Figure 5:**
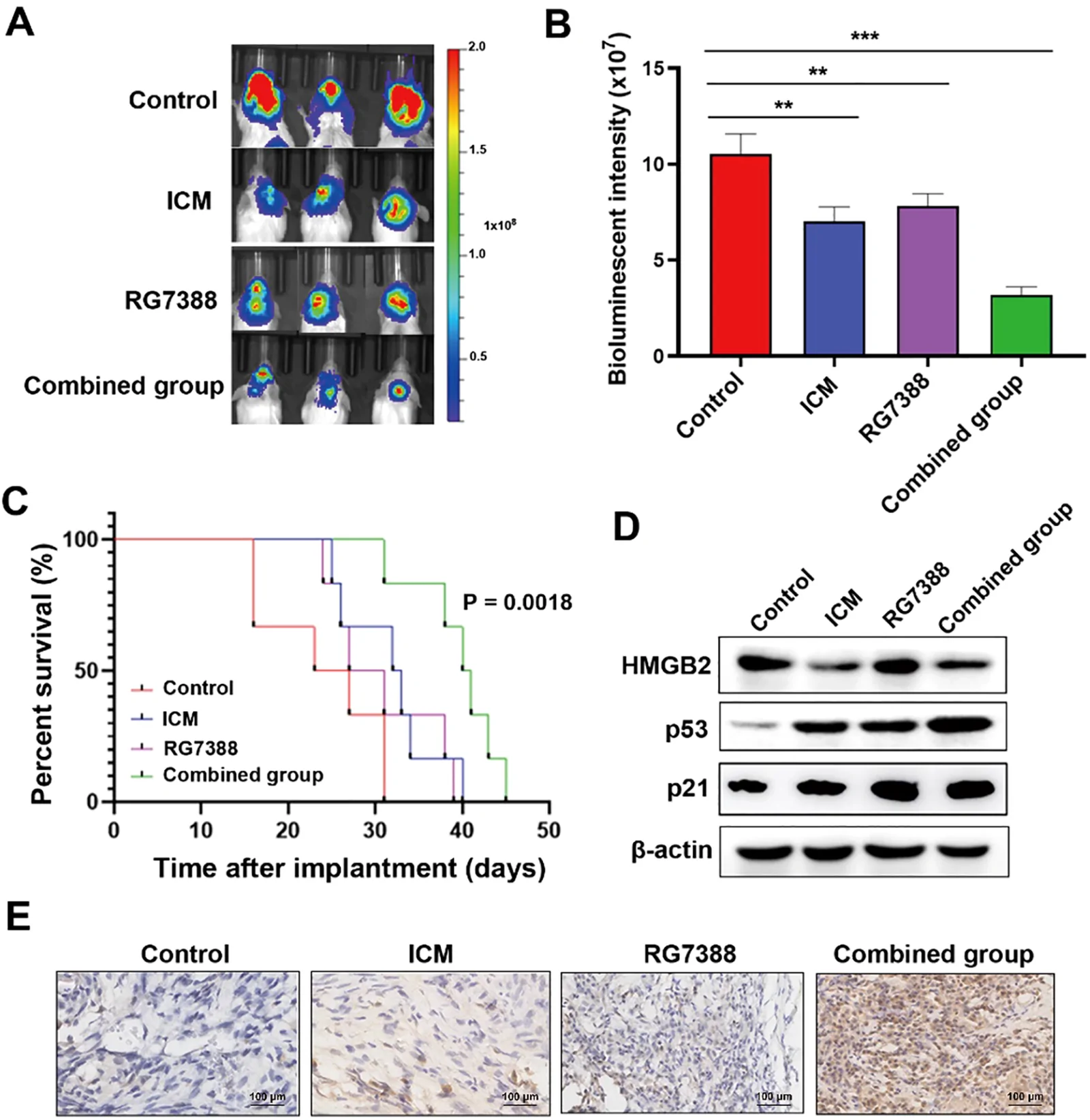
The effect of HMGB2 on DIPG tumor growth *in vivo*. **(A)** Representative bioluminescence images after generating orthotopic DIPG xenograft model by injecting luciferase-transduced DIPG cells into the mouse pons. Bioluminescent imaging was conducted on the 21^st^ day after cell implantation. **(B)** Quantification of bioluminescent intensity of mice from different groups (n=5 per each group). One-way ANOVA followed by *post-hoc* multiple comparison tests; **P < 0.01, ***P < 0.001. **(C)** The survival of mice bearing DIPG tumor was performed using Log-rank survival analysis. The mice were treated with HMGB2 inhibitor ICM (10 mg/Kg/day) and/or RG7388 (50 mg/Kg/day). The median survival of each group: Control group: 28 days; ICM group: 32 days; RG7388 group: 30 days; Combination group: 41 days. Pairwise Log-rank test statistics: Control group vs ICM group: P = 0.042; Control group vs RG7388 group: P = 0.071; Control group vs Combined group: P = 0.0018; ICM group vs Combined group: P = 0.0025; RG7388 vs Combination: P = 0.0011. **(D)** Tumor tissues from different treatment groups were collected for western blot analysis. **(E)** Representative images after immunohistochemical staining of p21 in tumor tissues from different groups.

## 4 Discussion

Recent studies have increasingly focused on the role of HMGB2 in cancer biology, identifying it as a potential oncogene in several cancer types. Research suggests that overexpression of HMGB2 promotes cancer cell proliferation, metastasis, and resistance to apoptosis (11, 14, 15, 27). However, its role in DIPG tumorigenesis remains unclear. In the current study, we conducted comprehensive analysis of HMGB2 function across pan-cancers, examining its gene expression levels and prognostic value. This research indicated that HMGB2 may act as an oncogene in tumor development and progression. Overexpression of HMGB2 was observed in DIPG cell lines, and its knockdown significantly inhibited DIPG cell proliferation ability and induced cell apoptosis. Additionally, we found that HMGB2 inhibition activated the p53 signaling pathway, as shown by bulk RNA sequencing analysis. This finding was further confirmed by an increase in p53 expression in HMGB2-knockdown DIPG cells. Moreover, the combination of HMGB2 inhibition with the p53-MDM2 inhibitor RG7388 synergistically suppressed DIPG cell growth by activating p53/p21 signaling pathway and thus promoting cell apoptosis *in vitro* and *in vivo*. These results suggest that combining HMGB2 with MDM2 inhibitors may offer a potential direction for future clinical translational research.

Most DIPGs carry mutations in histones 3.3 and 3.1, making these primary mutations potential therapeutic targets. Encouraging preclinical studies have shown promising results with GSK-J4, a JMJD3 demethylase inhibitor, and Panobinostat, a histone deacetylase inhibitor, that target H3K27M (28, 29). Other common mutations in DIPG include mutations TP53 (30), PDGFRA (31), EGFR, and ACVR1 (32), which also offer potential therapeutic targets. These preclinical trials have demonstrated anti-tumor properties in DIPG treatment. However, their narrow therapeutic index and dose-limiting toxicities often hinder their effectiveness. In addition, due to DIPG’s complex genetic landscape, combination therapies are often considered in the clinical settings for improving long-term survival (30, 32–34). In this study, we demonstrate that HMGB2 was overexpressed in DIPG cells, and inhibition of HMGB2 suppressed DIPG cell proliferation and induced cell apoptosis by activating p53/p21 signaling pathway, suggesting HMGB2 could serve as a valuable therapeutic target for DIPG.

HMGB2 binds to DNA and regulates transcriptional activity, playing a crucial role in maintaining genomic stability. In cancers such as lung cancer (35), breast cancer (15), hepatocellular cancer (36), and pancreatic cancer (37). HMGB2 expression is often elevated, promoting tumor cell survival and enhancing tumorigenic pathways. He et al. found that down-regulation of HMGB2 inhibited proliferation and invasion of renal tumor cells via inhibition of p-38MAPK pathway (38). In addition, microRNA-130a was reported to regulate downstream of oncogene HMGB2 expression to promote glioma growth (39). Therefore, targeting HMGB2 to reduce its overexpression has been shown as a promising therapeutic strategy in some cancers. Studies have shown that silencing HMGB2 expression can sensitize tumor cells to chemotherapy and radiotherapy by disrupting DNA repair mechanisms and cell cycle progression (11, 13, 40). Syed et al. demonstrated that HMGB2 inhibition could enhance the efficacy of existing chemotherapeutic regimens (cisplatin and 5-fluorouracil) for treatment of head and neck squamous cell carcinoma (41). Inflachromene (ICM) is a novel small molecule inhibitor that targets HMGB2 by disrupting its post-translational modifications and preventing its translocation to the cytoplasm (19). Preclinical studies have demonstrated that ICM exhibited significant anti-inflammatory efficacy in microglia-mediated neuroinflammation, as well as potential antiepileptic effects in mouse epilepsy models (19, 42). Here, we found the combination of ICM and MDM2 inhibitor demonstrated synergistic therapeutic effects in the treatment of DIPG, which lays the foundation for further exploration of clinically applicable combination therapies.

Some limitations are shown in this study. First, the molecular mechanism underlying HMGB2 silencing-mediated p53 upregulation was not explored in the current research. Existing research findings suggest that the regulation of p53 by HMGB2 is more likely an indirect regulatory mode. For instance, HMGB2 may regulate the ubiquitin-dependent degradation process of p53, thereby affecting its expression level (25, 26, 38). Second, HMGB2 can participate in tumor progression by activating pathways such as MAPK and PI3K/Akt (38, 39), and these pathways have been confirmed to indirectly regulate the stability of the MDM2-p53 complex (23, 30). It is speculated that HMGB2 may indirectly influence the degradation process of p53 by regulating the activity of such pathways. A limitation of this study is that the above speculations have not been verified through experiments. In the future, we will focus on conducting mechanistic verification around directions such as “p53 ubiquitination modification”, “interaction between HMGB2 and MDM2”, and “mediating role of downstream signaling pathways” to clarify the regulatory network between the two. Third, additional studies are needed to address the safety, efficacy, and long-term effects of combining HMGB2 inhibition with a p53-MDM2 inhibitor. Fourth, this study lacks data on ICM’s blood-brain barrier penetration, pharmacokinetics, and safety in pediatric tumors, and its clinical translation requires further verification. Finally, the potential benefits of combining HMGB2 inhibition with other therapeutic strategies for DIPG should be further explored to support the case for future clinical trials.

Nevertheless, this study has several inevitable limitations. First, ICM is a dual-target inhibitor for both HMGB1 and HMGB2, and we could not fully exclude the potential function of HMGB1 in DIPG. Combined with shRNA-mediated specific HMGB2 knockdown results, we confirmed that HMGB2 plays a critical oncogenic role in DIPG. Second, we only detected p53 total protein level and its downstream p21, while p53 transcriptional activity, post-translational modification and other target genes were not explored, the detailed mechanism of p53 regulation needs further research. Third, we verified that all DIPG cell lines used in this study were TP53 wild-type, so the current combination strategy may not be applicable to TP53-mutant DIPG subtypes. Fourth, the blood-brain barrier penetration efficiency and pharmacokinetics of ICM have not been quantitatively detected; although *in vivo* experiments confirmed its anti-tumor effect in mouse orthotopic model, clinical transformation needs further pharmacodynamic verification. Fifth, we did not perform immunohistochemical staining of HMGB2, p53 and Ki67 in tumor tissues, and the *in situ* protein expression and cell proliferation status remain to be verified. In addition, the sample size of animal experiments (n=5 per group) is acceptable for preclinical basic research, and larger sample cohorts are needed in future studies.

In conclusion, the current study demonstrated that suppression of HMGB2 inhibits DIPG cell proliferation and promotes cell apoptosis. This study reports the first time that inhibition of HMGB2 was associated with activation of p53 signaling pathway. Combination treatment with HMGB2 and MDM2 inhibitors may represent a promising preclinical strategy warranting further investigation for DIPG treatment.

## Data Availability

The raw sequence data reported in this paper have been deposited in the Genome Sequence Archive (Genomics, Proteomics & Bioinformatics 2021) in National Genomics Data Center (Nucleic Acids Res 2024), China National Center for Bioinfomation/Beiling Institute of Genomics, Chinese Academy of Sciences (BioProject: PRJCA037584) that are publicly accessible at https://ngdc.cncb.ac.cn/search/specific?db=bioproject&q=PRJCA037584.

## Supplementary material

Supplementary Figure 1 Quantitative analysis for both IHC and Western blot data of the tumor tissues. (A) Quantitative analysis for Western blot data of the tumor tissues from different treatment groups. One-way ANOVA followed by *post-hoc* multiple comparison tests; **P < 0.01, ***P < 0.001. (B) Quantitative analysis of p21 for IHC of the tumor tissues from different treatment groups. One-way ANOVA followed by *post-hoc* multiple comparison tests; **P < 0.01, ***P < 0.001.
